# Endoscopically controlled surgery with open hemilaminectomy for the treatment of intradural extramedullary tumors: an operative technique and short-term outcomes of 20 consecutive cases

**DOI:** 10.1186/s41016-020-00222-0

**Published:** 2021-01-04

**Authors:** Xiaorong Yan, Huiqing Wang, Cai Li, Yuanxiang Lin, Lin Lin, Shinong Zhu, Chenyang Wang, Zhangya Lin, Changzhen Jiang, Dezhi Kang

**Affiliations:** 1 Neurosurgery Department, The First Affiliated Hospital of Fujian Medical University, 20# Chazhong Road, Fuzhou City, Fujian Province China; 2 Neurosurgery Department, The First Affiliated Hospital of University of South China, Hengyang City, Hunan Province China; 3 Operation Room, The First Affiliated Hospital of Fujian Medical University, Fuzhou City, Fujian Province China; 4 Neurosurgery Department, Jinjiang Hospital Jinnan Branch Courts, Jinjiang City, Fujian Province China

**Keywords:** Endoscopically controlled surgery, Open hemilaminectomy, Intradural extramedullary tumors

## Abstract

**Background:**

To present a surgical technique for the treatment of intradural extramedullary (IDEM) tumors by using endoscopically controlled surgery with open hemilaminectomy technique.

**Methods:**

In this study, 20 patients with 22 IDEM tumors were enrolled. An endoscopically controlled surgery with open hemilaminectomy was employed to remove the tumors. Data related to clinical symptoms and medical images before and after surgery were collected for perioperative evaluation and follow-up analysis.

**Results:**

All the tumors in 20 patients were well removed. The clinical symptoms were significantly reduced in all the patients as well. The short-term follow-up data showed that there was no tumor recurrence or spinal deformity.

**Conclusion:**

The endoscopically controlled surgery with open hemilaminectomy technique provided favorable exposure and satisfactory resection to the IDEM tumors. It may be an effective surgical method for treating IDEM tumors. Larger samples and longer follow-up data are needed to verify its long-term effectiveness.

## Background

The hemilaminectomy approach has been taken as an effective method for treating intradural extramedullary (IDEM) spinal tumors [1–3]. Compared with laminectomy approach, hemilaminectomy can retain more ligaments and bone structures to reduce the risk of postoperative spinal instability and preserve the contralateral paravertebral muscles to alleviate postoperative pain [1]. Nevertheless, hemilaminectomy contains a number of disadvantages in terms of surgically dissecting a number of lesions, such as ventrally located tumors, bilateral extension lesions in the spinal canal, and tumors that are densely adherent to the cord surface. In these situations, direct and adequate visualization is essential for safe dissection, while avoids injury to the delicate spinal cord [4].

Endoscopic techniques in neurosurgery have offered superior visualizing deep lesions with less retraction and invasion of important functional structures [5, 6]. To achieve an appropriate surgical exposure and reduce retracting to the spinal cord during surgery, we attempted to perform an endoscopic technique in our institute. During the past 2.5 years, 20 patients with IDEM spinal tumors underwent endoscopically controlled surgery with open hemilaminectomy technique. In the present study, we reported the short-term outcomes of those patients who underwent endoscopically controlled surgery with open hemilaminectomy technique.

## Methods

### Patients

We retrospectively reviewed 20 patients, including 12 women and 8 men who were admitted to the Department of Neurosurgery of the First Affiliated Hospital of Fujian Medical University (Fuzhou, China) between July 2016 and December 2018. Those patients were diagnosed with intraspinal tumors confirmed by magnetic resonance imaging (MRI). All patients met the inclusion criteria, in which the tumor was located in the spinal canal, and agreed to undergo endoscopically controlled surgery with open hemilaminectomy technique. The study protocol was approved by the Ethics Committees of the First Affiliated Hospital of Fujian Medical University. We excluded the recurrent cases for the incomplete bone structure and possible artificial implants. Patients with spinal tumors concomitant herniated disk were excluded from the study as well. We also excluded the lesions at the level of the filum terminale/cauda equine.

### Endoscopically controlled surgery with open hemilaminectomy

After undergoing general anesthesia, the patient was placed in a prone position. Intraoperative neurophysiological monitoring of motor-evoked potentials was applied to indicate the retraction of the spinal cord and neurological function. A straight midline skin incision was performed. Then, a modified hemilaminectomy was carried out to make a surgical corridor. Briefly, the paraspinal muscle was fully dissected and retracted by spring hooks fasten to a fixed arm, which was analogous to Tola et al.’s study, aiming to eliminate any external hindrance [7]. A neurosurgical endoscope was then introduced. The surgeons stood on one side of the patient, while the monitor was placed oppositely. A high-speed drill was utilized under an endoscopic view to partially remove the lamina and the base of the spinous process.

**Fig. 1:**
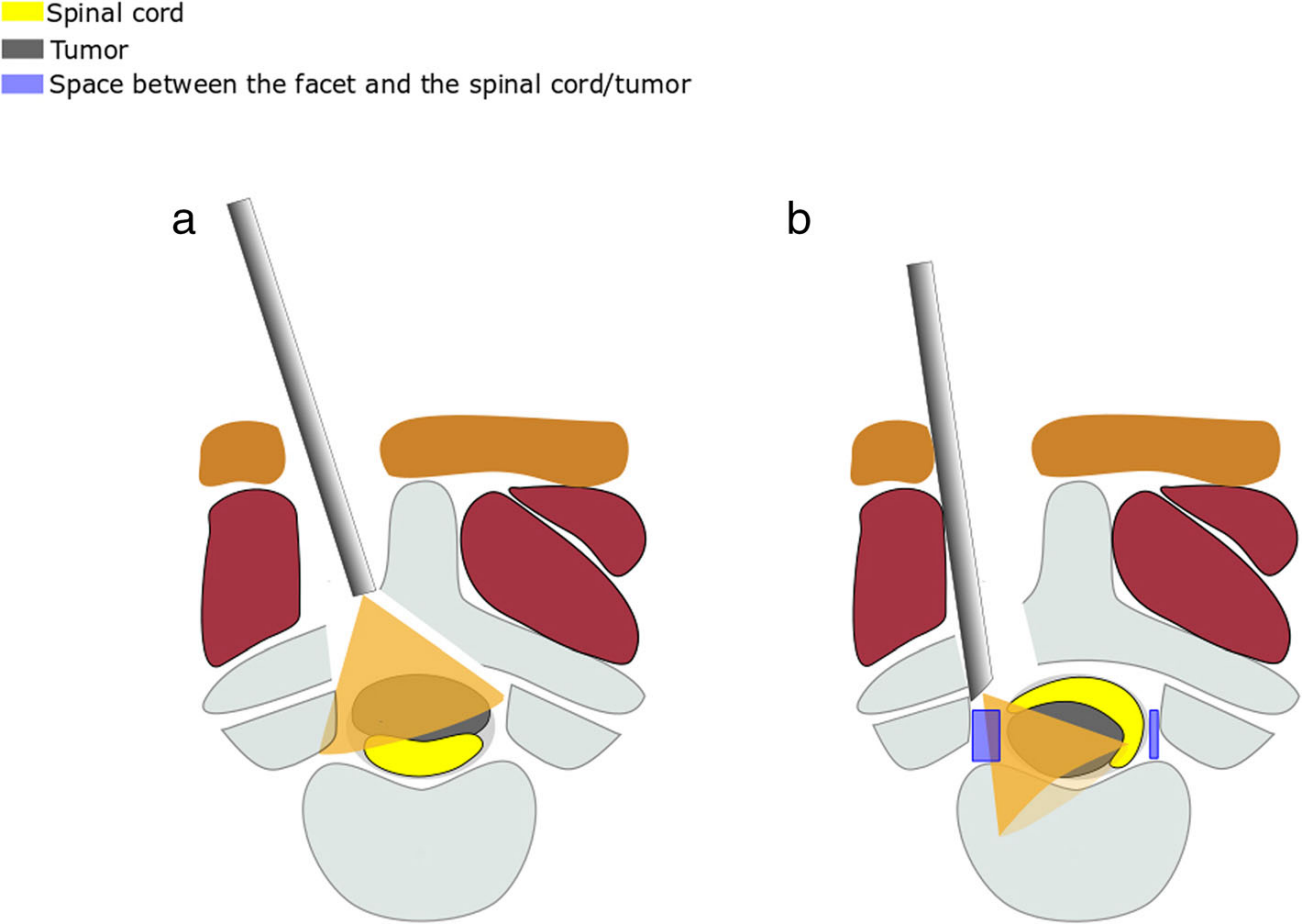
Schematic diagram of endoscopic surgery with the hemilaminectomy approach. **a** As the basement of the spinous process was partially removed, the endoscope could provide a satisfactory surgical view for the exposure of the dorsal tumor and the spinal canal. **b** After a limited excision of the facet joint, the space between the facet and the spine cord/tumor was enlarged, which could help to provide a superior endoscopic view for the tumor exposure (the purple part represents the space between the facet and the spine cord/tumor)

For a large dorsally located tumor, we extended the drilling of the base of spinous process to ensure that the endoscopic view could cover the entire tumor (Fig. 1a). For ventral tumor, a limited removal of the facet joint may be essential to achieve a perfect endoscopic view for the ventral or contralateral space of the spinal canal (Fig. 1b). Then, the dura was sharply opened by a knife and scissor, and the dural edge was retracted by suture to the paravertebral muscles.

**Fig. 2:**
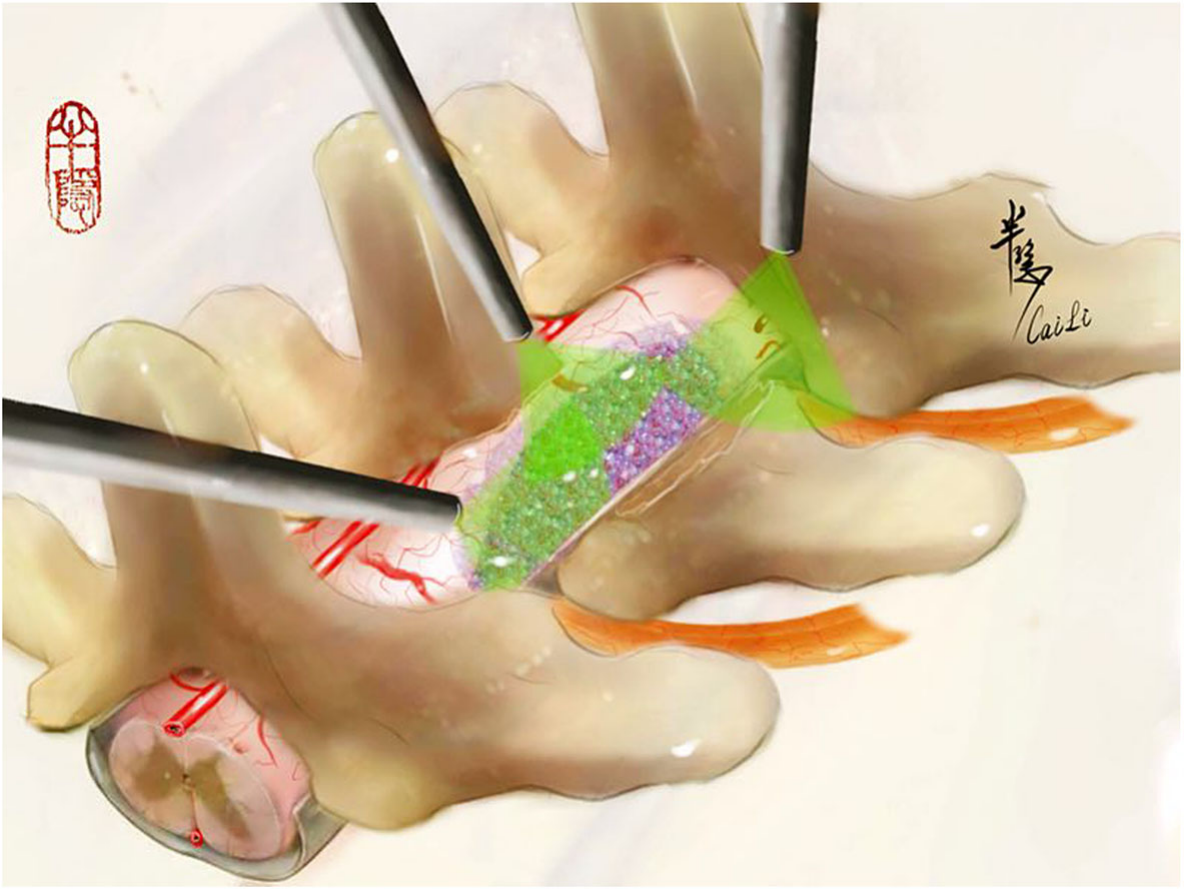
Simulated diagram showing the different placement directions of an endoscope to observe the lesion (the purple part represents the lesion)

Under endoscopic view, microsurgical skills were adopted. The extracapsular dissection of the tumor away from the adjacent cord combined with the intracapsular debulking technique was used to deal with the large tumor. Ultrasonic aspirator was employed for tumor debulking if necessary. In the process of tumor resection, the tumor can be observed from different orientations by adjusting the placement angle of the endoscope under an open surgical field (Fig. 2).

After tumor resection, a 45- or 0-degree rigid endoscope (Karl Storz, Tuttlingen, Germany) was placed intradurally to visualize the canal to identify a residual tumor. Eventually, we sutured the margin of the dura and cover the dura with DuraGen (Integra LifeSciences Corp., Plainsboro Township, NJ, USA). According to the extent of the dura defect, in necessity, the tension-reduced duraplasty was undertaken to ensure that there is no dural stenosis. In case #6, a spinal meningioma was fully originated from the central portion of the ventrally spinal dura, the tumor-attached dura was removed without any patch in the site. The dense posterior longitudinal ligament prevents the cerebrospinal fluid leakage. For cases with ventrolateral spinal meningioma, we only performed extensive cauterization to the tumor-attached dura without dura resection, and also conducted tension-reduced duraplasty with watertight suture to avoid possible dural stenosis and cerebrospinal fluid leakage.

### Study design

The medical records and follow-up notes of 20 patients were reviewed. The tumor location was defined by preoperative MRI. The extent of tumor resection was according to surgical outcomes and evaluated by postsurgical MRI within 72 h. The preoperative functional state of the spinal cord and surgical outcome was assessed by the American spinal injury association impairment scale (AIS), and the visual analog scale (VAS) was applied to determine the extent of preoperative and postoperative pain. AIS and VAS were assessed by taking the average score given by 2 physicians (ZSN and YXR) of the patient on admission. If a large deviation was seen, the final score was determined by a third physician (JCZ). Any complications associated with the surgery were recorded as well.

### Statistics analysis

The statistical analyses were performed with the SPSS software (version 21.0 for Windows). Student’s *t* tests were applied to assess VAS score which was used to evaluate the extent of preoperative and postoperative pain. Differences were considered significant at a *P* value of < 0.05.

## Results

### Patients’ clinical characteristics

In the present study, the patients’ mean age was 42.30 ± 14.16 years, which ranged from 23 to 67 years (Table 1). The mean duration of follow-up was 20.30 ± 5.02 months. The main clinical presentation included pain, sensory deficit, and limb weakness. As presented in Table 2, astriction and dysuria were observed in 4 patients. Preoperative MRI showed that all tumors were located intradurally, including 16 patients with tumor at the thoracic level, and 4 patients with tumor at lumbar or sacral level. Besides, 11 patients had tumors that extended to two metameric levels: 6 patients had tumors limited to one level, 2 patients had tumors extended to 3 levels. In addition, 1 patient with neurofibromatosis-2 (NF-2) had multiple lesions in the spinal canal, and we resected 3 lesions with extension from T9-T12. Considering the location of the tumors, 10 lesions in 8 patients were ventrolateral to the spinal cord, 7 patients had posterior tumors, 2 patients had lateral and ventral tumors, respectively, and 1 patient had posteriolateral tumors (Table 1).

**Table 1:** Patient characteristics, surgical information and outcome evaluation

Patient	Sex	Age	Level	Location	Resection of Simpson grade	Surgery duration (h)	Blood loss (mL)	Pathology
1	Female	67	L4	Ventrolateral		3.5	200	Schwannoma
2	Male	67	T12	Lateral		2.8	200	Schwannoma
3	Female	54	T7-T8	Ventrolateral	II	5.5	400	Meningioma
4	Female	45	T11-T12	Posteriorly		4.3	200	Schwannoma
5	Female	49	T5-T6	Posteriolateral	I	5	200	Meningioma
6	Female	23	T2-T3	Ventral	I	4	200	Meningioma
7	Female	42	T6-T7	Ventrolateral		2.5	300	Schwannoma
8	Male	47	L5-S1	Lateral		4.5	300	Schwannoma
9	Male	58	T10	Ventrolateral		3	100	Schwannoma
10^a^	Female	29	T9-T12	Ventrolateral		6.5	600	Neurofibromatosis
11	Female	33	T5	Ventrolateral	II	2.4	200	Meningioma
12	Female	24	T2-T4	Posteriorly	II	3.5	200	Meningioma
13	Male	47	T11-L1	Ventral		4	300	Schwannoma
14	Male	43	L4-L5	Posteriorly		2.2	100	Schwannoma
15	Male	28	T11-T12	Posteriorly		3.0	200	Schwannoma
16	Female	34	T3-T4	Ventrolateral	II	4.5	250	Meningioma
17	Female	43	T9-T10	Posteriorly		2.5	300	Schwannoma
18	Female	37	L4-L5	Ventrolateral		2.5	150	Schwannoma
19	Male	58	T2	Posteriorly		3.0	300	Meningioma
20	Male	18	T9	Posteriorly		2.5	200	Schwannoma

^a^The patient with NF-2 had 3 lesions extending from T9-T12

**Table 2:** Patient symptoms

Patient	Symptoms				
	Pain	Sensory deficit	Limb weakness	Astriction	Dysuria
1	√				
2	√				
3	√	√	√		
4	√	√	√		
5		√	√		
6	√	√	√	√	√
7	√				
8	√	√	√		
9	√	√	√		
10	√	√	√	√	√
11	√				
12	√	√	√		
13	√	√	√	√	√
14	√	√			
15	√	√	√		
16	√	√	√	√	√
17	√	√	√		
18	√	√			
19	√	√	√		
20	√	√	√		

### Surgical outcomes

**Fig. 3:**
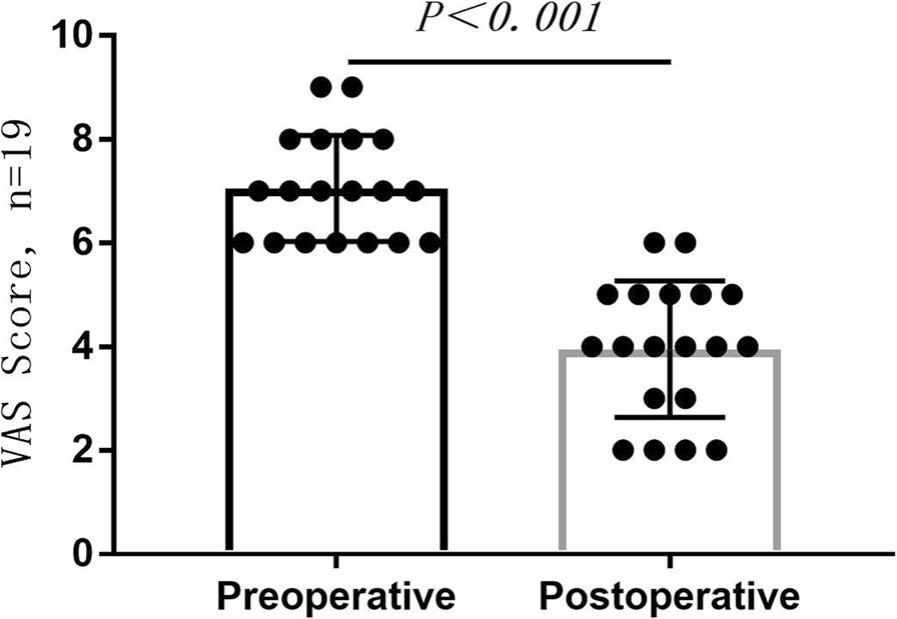
Preoperative and postoperative evaluation of VAS for patients who had a preoperative pain. Evaluation of VAS showed that the pain was relieved in each patient. The paired *t* test demonstrated that there was a significant decline in postoperative VAS (*P* < 0.001)

Monosegmental hemilaminectomy was efficient to create a surgical corridor for tumor resection in all patients, except for a patient with 3 lesions. With the help of an endoscope, all the tumors were fully removed, involving Simpson grade I and grade II resection according to surgical outcomes and postoperative MRI. Furthermore, transient decline of intraoperative motor-evoked potentials was observed in only 2 patients, who both recovered by stopping surgery for 5 min. The average duration of surgery was 3.58 ± 1.18 h, which ranged from 2.2 to 6.5 h. The most time-consuming surgery was to resect 3 lesions in the patient with NF-2, resulting in maximum blood loss (Table 1). Pathological diagnosis confirmed 12 schwannoma (60.00%), 7 meningioma (35.00%), and 1 neurofibromatosis. With respect to surgical outcomes, there was no deterioration of AIS in any patient, and some outcomes were improved at the late follow-up (Table 3). The results of VAS showed that all the patients, who had preoperative pain, had a decrease in pain on the day of discharge. There was no unplanned secondary surgery, post-operative blood, or CSF leakage in this series. In addition, the paired *t* test disclosed that there was a significant decrease in postoperative VAS (*P* < 0.001) (Fig. 3). The latest follow-up postoperative 3D CT scan images showed that all the patients had neither spinal malformation nor deformity.

**Table 3:** American spinal injury association impairment scale (AIS)

Patient	Preoperative	Discharge	Latest follow-up
1	E	E	E
2	E	E	E
3	D	D	D
4	D	D	D
5	D	D	E
6	C	D	D
7	E	E	E
8	D	D	E
9	D	D	D
10	B	B	B
11	E	E	E
12	D	D	E
13	D	D	D
14	E	E	E
15	C	C	D
16	C	C	D
17	B	B	C
18	E	E	E
19	D	D	D
20	E	E	E

### Illustrative cases

#### Case #1

**Fig. 4:**
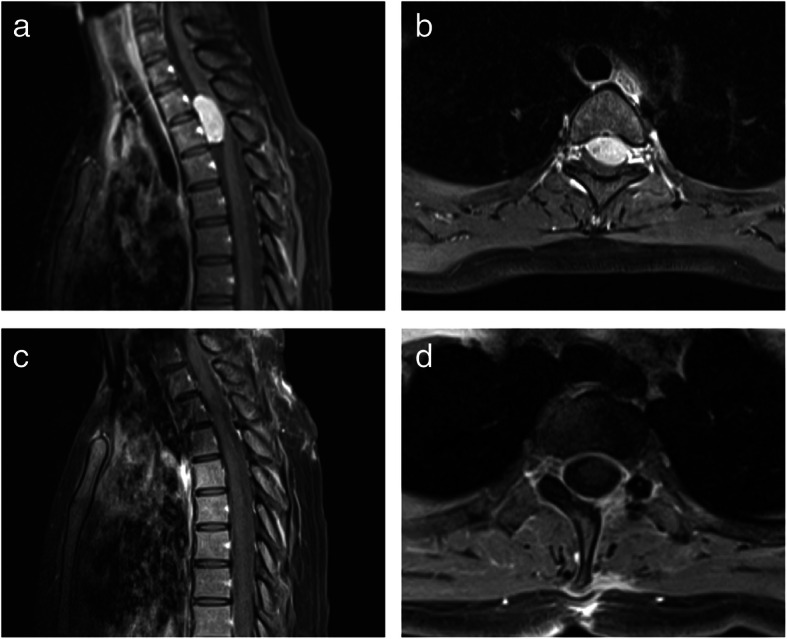
Preoperative and postoperative MR images of the illustrative case #1. **a**, **b** Sagittal and axial preoperative T1-weighted gadolinium-enhanced MR images revealed that a ventrally located intradural extramedullary tumor at the level of T2-T3. **c**, **d** Sagittal and axial preoperative T1-weighted gadolinium-enhanced MR images showed that there was no residual tumor

**Fig. 5:**
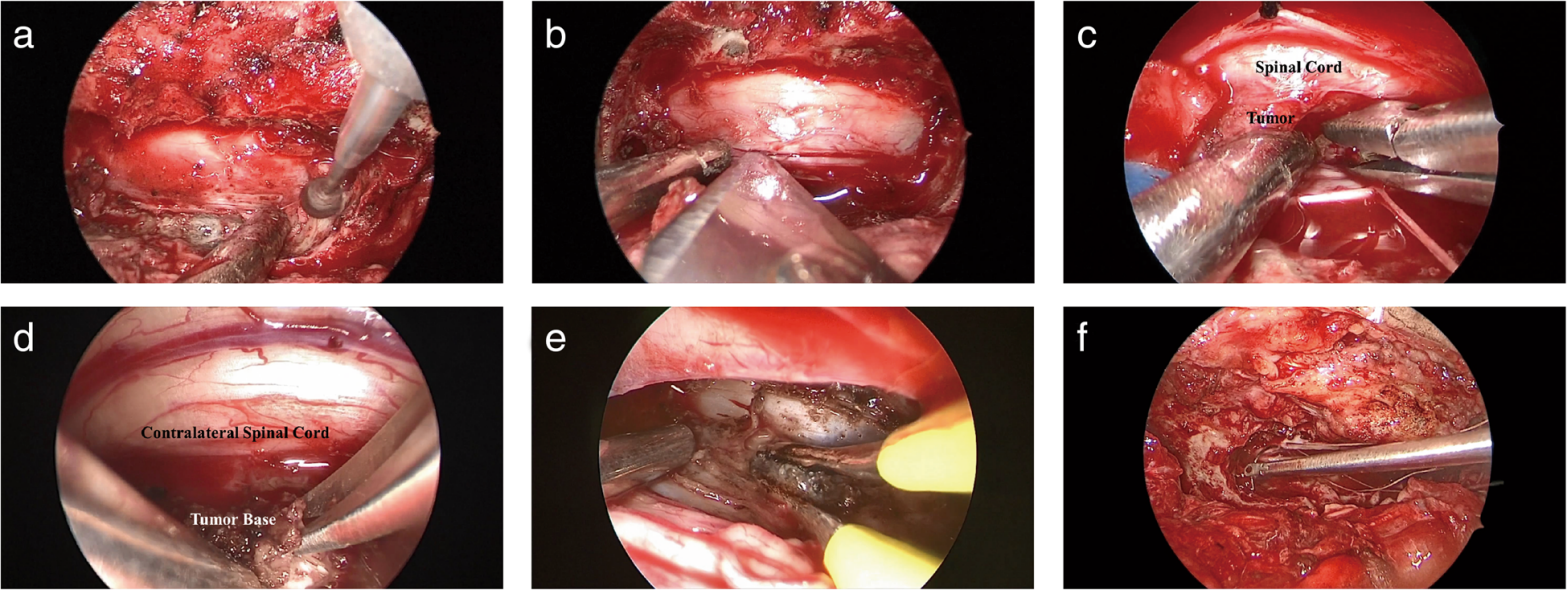
Intra-operative photographs of an endoscopic surgery with the hemilaminectomy approach: a patient (illustrative case 1) with a totally ventral meningioma. **a** Drilling the base of the spinous process. **b** Dura was opened by a scalpel. **c** Tumor debulking under an endoscopic view without retracting of spinal cord. **d** A 45° endoscope was employed to observe the central (tumor base) and contralateral parts (spinal cord) of the canal when cutting the tumor base. **e** Coagulating the residual tumor originating from dura. **f** Suturing the dura

She (patient no. 6) was a 23-year-old woman with an intradural ventral tumor. This patient had lower limb weakness for 8 months before admission, along with back pain with a VAS of 6, sensory deficit, astriction, and dysuria. Neurologic examination showed grade 4 of muscle strength in both lower limbs, a slight hypalgesia below level T4, and decreased vibratory sense in both lower extremities. Contrast-enhanced MRI of the spine showed a lesion located ventrally to the spinal cord at the level of T2-T3 (Fig. 4). The mass had intense and homogeneous enhancement. On the basis of the location of the mass, the surgical challenge was how to expose the tumor and reduce retraction of the spinal cord. Thus, we employed endoscopy to expand the surgical view of the tumor, as described in the “Methods” section (Fig. 5). With the help of endoscopy, the ventral mass was debulked and completely removed with minimal spinal cord retraction. The postoperatively MRI displayed complete tumor removal (Fig. 4). Pathological examination uncovered that the mentioned lesion was a meningioma. Limb weakness and sensory deficit were resolved at 3-month of follow-up.

#### Case #2

**Fig. 6:**
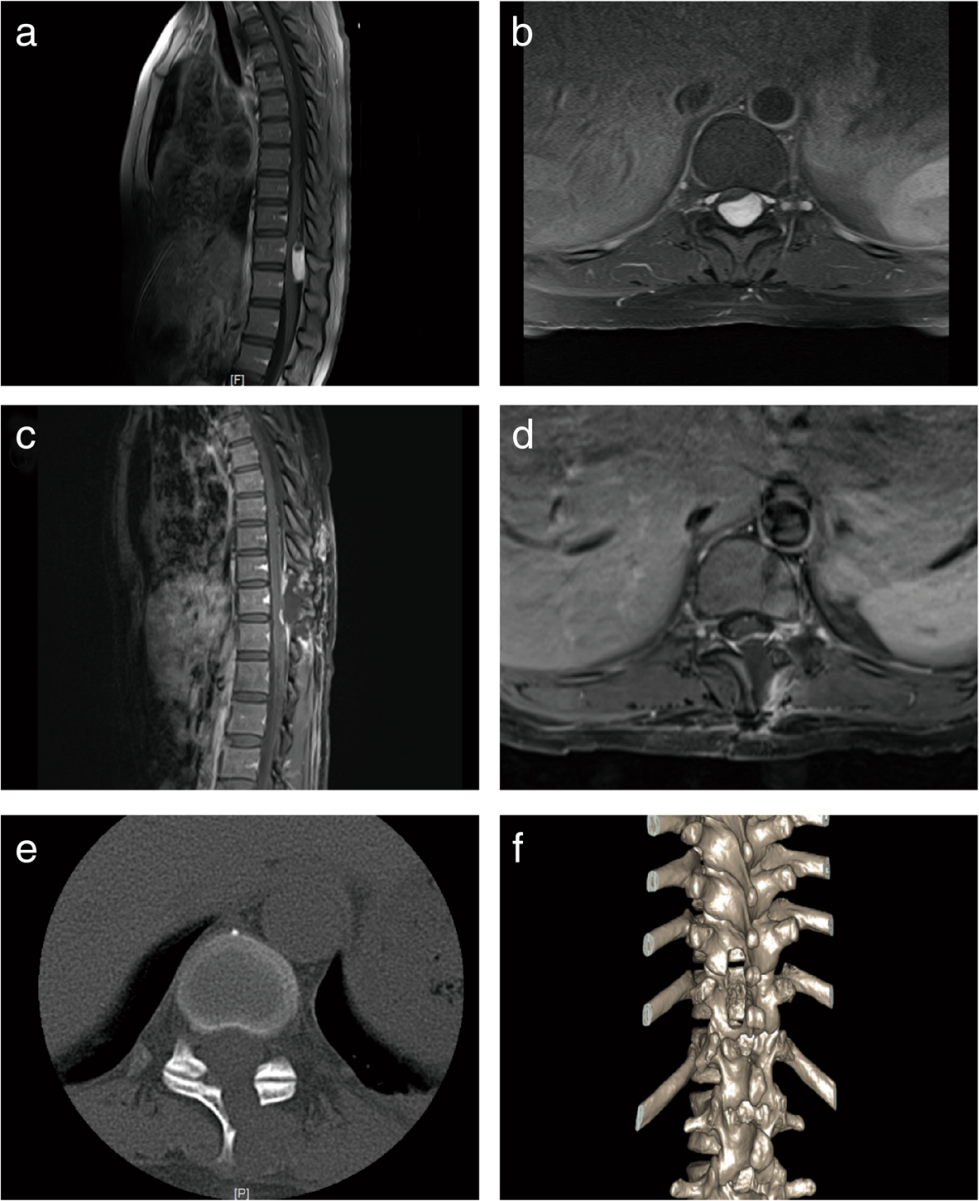
Preoperative and postoperative MR images of the illustrative case #2. **a**, **b** Sagittal and axial preoperative T1-weighted gadolinium-enhanced MR images revealed that a posteriorly located intradural extramedullary tumor at level of T11-T12. **c**, **d** Sagittal and axial preoperative T1-weighted gadolinium-enhanced MR images displayed that the tumor was fully removed. **e**, **f** Axial CT and three-dimensional CT presented the integrity of the facet joints with no spine malformation

She (patient no. 4) was a 45-year-old woman with limb weakness and sensory deficit in her left limb accompanied with back pain for 2 months. Contrast-enhanced MRI of the spine illustrated a lesion located posteriorly to the spinal cord at the level of T11-T12 (Fig. 6). This patient underwent endoscopically controlled surgery with open hemilaminectomy. The postoperative MRI showed that the tumor was totally removed, and computed tomography (CT) scan depicted the integrity of the facet joints, which are important to the stability of the spine (Fig. 6). This operation relieved the pain postoperatively and the limb weakness and sensory deficit were resolved at 6-month of follow-up. Pathological examinations demonstrated that the tumor was a schwannoma.

## Discussion

The stability of the spine and the exposure of the mass are a delicate balance for surgeons. Total single and/or multi-level laminectomy/laminotomy has been widely used for the resection of spinal lesions, since full exposure of the cord and tumors could be achieved. However, the disadvantages of laminectomy/laminotomy are related to the destruction of the bilateral paraspinal muscle and removal of the posterior spinal elements, which may cause more postoperative complications and spinal instabilities [8].

Recently, hemilaminectomy with microsurgery has become a promising technique for the treatment of a number of spinal tumors. This approach preserves the spinous process and its base, as well as the contralateral lamina, including ligamentum flavum and muscle [9]. Nevertheless, surgical reports demonstrated its preferred use for small and laterally located masses. Several previous studies have attempted to improve various aspects of bone work required during hemilaminectomy to gain more exposure for the tumor resection [3, 9, 10].

With emerging state-of-the-art technologies, at the current era, endoscopic surgery is considered to have a higher resolution and definition view than previous decades [11]. These advances accompanied with the flexible placement through surgical corridor allow surgeons to have more direct angles and clearer display to identify the important structure and the nerve-tumor interfaces. These changes may not only improve the tumor resection but also reduce the residual tumor [12, 13].

In this consecutive endoscopic surgery for ventrolaterally and ventrally located tumors, with a limited removal of the articular process, the tumor exposure is facile. By using a 45-degree angle endoscope, the contralateral side of the spinal canal could be visualized with little hindrance, as well as removing the ventral tumor from one side to the other under a wide and direct view. Hence, the manipulation or traction of the spinal cord could be reduced as much as possible. Moreover, by adjusting the placement angle of the endoscope under an open surgical field, the lesion could be closely observed from different orientations (Fig. 2). This might be helpful to find nerve-tumor interface under a more direct angle and avoid unnecessary damage to the nerve. A widely open endoscopic view using the bimanual technique would also help surgeons reach the ventral dura mater, where meningiomas originate.

For large tumors located posteriorly to the spinal cord, laminectomy was taken as an appropriate choice into account compared with hemilaminectomy, because operations under the latter approach might not have a favorable view to the contralateral part of the canal, which might cause damage to the nerve during the tumor dissection. In our procedure, with partial resection of the base of the spinous process, the wide-angle view of the endoscope can be placed directly up to the tumor that is posterior to the spinal cord and provides a full view (Fig. 1a).

In terms of decreasing residual tumor, Al-mefty et al. reported that the subsequent use of an endoscope could effectively help to find out the tumor remnant and increase the total resection rate in brain tumor surgery [13]. In surgeries of the present study, after tumor removal, we observed the canal from multiple directions by adjusting the placement angle of the endoscope as we performed in the tumor resection procedure to indicate whether there is a residual tumor. As a result, in our short-term follow-up, no tumor recurrence was noted.

According to the postoperative MRIs, in the current research, all the patients had gross total resection of their tumors. In addition, all the patients had a favorable improvement of their pain on the day of discharge. Not only in the perioperative period, while in the late postoperative period, the patients sensed a decrease in pain.

Numerous studies have confirmed the spinal stability of hemilaminectomy surgery [14–16]. However, some authors have reported their experience in partially or totally removing the articular process for ventral masses, which may threaten the stability of the spine [17, 18]. In our series, with a panoramic view and close-up observation using the endoscope, the resection of the facet joint could be utmost reduced or avoided for a ventral or ventrolateral tumor. A laminectomy for a total dorsal meningioma was also avoided due to the favorable view obtained by the endoscope. The postoperative CT scan images and MRI findings showed that all the patients had neither malformation nor deformity in a short-term follow-up.

In addition, in the current research, we did not use a conventional metal retractor in our procedure due to its stiffness and thickness, which may cause resistance for the endoscope. Alternatively, we used a spring hook fastened to an external fixed mount. With the help of this technique, the endoscope rod can lean directly on the incision margin, allowing a wider angle of inclination for the endoscope to reach the tumor.

The present study contains a number of limitations. This is a retrospective study of a single surgeon’s experience. Meanwhile, we did not set the control groups and compare the postoperative outcomes and clinical data with hemilaminectomy under microsurgery or tubular retractor surgery. The length of our surgical incision was not different from general microsurgery, and that was remarkably longer than tubular retractor surgery. Actually, our procedure was just a preliminary attempt to use an endoscope in removing IDEM tumors in our center. We were trying to find the feasibility of this technique in spinal tumors and not seeking to declare an absolute predominance of this technique comparing with the traditional surgeries under a microscope.

For spinal tumor surgery, an adequate exposure is of great importance for tumor resection and nerve protection [4]. Researches in microsurgery through hemilaminectomy have concerned about the exposure of the spinal canal by focusing on the points including degrees of lateral bone resection, dentate ligament division, and degree of cord rotation. Although, in general, the traditional microscopic surgeries may be adequate for the exposure of the surgical field in dealing with tumors occupying both sides of the dura sac and/or tumor ventrally located to the spinal cord. Surgeons may extend the bone resection, slope of operation bed, rotate the cord, or even use the laminectomy instead to solve the problem of inadequate exposure. These may influence the outcome of the surgery and cause potential risks to functional protection. Our procedure takes the advantage of endoscopic close-up and panoramic visualization to promote the exposure of the surgical field. Moreover, the tumor can be observed from different orientations by adjusting the placement angle of the endoscope under an open surgical field (Fig. 2). Our preliminary attempt was to combine the advantages of endoscopic surgery with better visualization, flexibility of endoscope placement angle, and sufficient space of open surgery to achieve a superior tumor observation, nerve protection, and reduction of residual tumor. This technique may provide a reliable option for such situations when dealing with tumors expending to the contralateral spinal canal, and lesions are involved in multiple levels or a case that requires enough space for surgical manipulations.

## Conclusion

In conclusion, the results of the present study suggest that IDEM neoplasms can be safely and effectively treated with endoscopically controlled surgery with open hemilaminectomy technique. The use of an endoscope can provide a sufficient exposure for the surgical field, while reduce spinal-cord manipulation without unnecessary bone reduction. Larger samples and longer follow-up data are required to verify its long-term effectiveness.

## Data Availability

The datasets used and/or analyzed during the current study are available from the corresponding author on reasonable request.

## Abbreviations

IDEM: Intradural extramedullary
MRI: Magnetic resonance imaging
AIS: American spinal injury association impairment scale
VAS: Visual analog scale
NF-2: Neurofibromatosis-2
CT: Computed tomography
